# A Systematic Review of Pharmacological Treatment Options Used to Reduce Ischemia Reperfusion Injury in Rat Liver Transplantation

**DOI:** 10.1371/journal.pone.0122214

**Published:** 2015-04-28

**Authors:** Kenya Yamanaka, Philipp Houben, Helge Bruns, Daniel Schultze, Etsuro Hatano, Peter Schemmer

**Affiliations:** 1 Department of General and Transplant Surgery, University Hospital of Heidelberg, Heidelberg, Germany; 2 Department of Surgery, Graduate School of Medicine, Kyoto University, Kyoto, Japan; IDIBAPS - Hospital Clinic de Barcelona, SPAIN

## Abstract

**Background:**

Although animal studies models are frequently used for the purpose of attenuating ischemia reperfusion injury (IRI) in liver transplantation (LT), many of pharmacological agents have not become part of clinical routine.

**Methods:**

A search was performed using the PubMed database to identify agents, from which 58 articles containing 2700 rat LT procedures were selected. The identified pharmacological agents were categorized as follows: I - adenosine agonists, nitric oxide agonists, endothelin antagonists, and prostaglandins, II – Kupffer cell inactivator, III - complement inhibiter, IV - antioxidant, V - neutrophil inactivator, VI -anti-apoptosis agent, VII - heat shock protein and nuclear factor *kappa* B inducer, VIII - metabolic agent, IX - traditional Chinese medicine, and X - others. Meta-analysis using 7-day-survival rate was also performed with Mantel-Haenszel's Random effects model.

**Results:**

The categorization revealed that the rate of donor-treated experiments in each group was highest for agents from Group II (70%) and VII (71%), whereas it was higher for agents from Group V (83%) in the recipient-treated experiments. Furthermore, 90% of the experiments with agents in Group II provided 7-day-survival benefits. The Risk Ratio (RR) of the meta-analysis was 2.43 [95% CI: 1.88-3.14] with moderate heterogeneity. However, the RR of each of the studies was too model-dependent to be used in the search for the most promising pharmacological agent.

**Conclusion:**

With regard to hepatic IRI pathology, the categorization of agents of interest would be a first step in designing suitable multifactorial and pleiotropic approaches to develop pharmacological strategies.

## Introduction

Liver transplantation (LT) has been established as an effective therapy for end-stage liver disease and a standard surgical management option for hepatocellular carcinoma [1, 2]. Despite improvements in immunosuppressive protocols and surgical techniques, graft rejection episodes, as well as primary non-function (PNF) and primary delayed graft function (PDF) are still prevalent [3]. Ischemia Reperfusion Injury (IRI) is inevitable after LT and a major risk factor for PNF and PDF [4]. Furthermore, the shortage of organs available for LT has led to the increasing use of liver grafts with extended donor criteria (EDC) that have greater susceptibility to IRI [5].

Hepatic IRI occurs via a complex pathologic network that features a combination of factors, including impairment of sinusoidal endothelial cells (SECs), activation of Kupffer cells (KCs), disturbance of microcirculation, oxidative stress, inflammation, activation of complement factors, accumulation of leukocytes, apoptosis, and necrosis [6]. Some strategies that have been applied in experimental LT models to decrease IRI include the use of ischemic preconditioning, additives in preservation solutions, gene therapy, and the application of numerous pharmacological agents [7]. From the point of clinical application, various experimental studies have focused on developing pharmacological strategies to reduce PDF and PNF with the aim of disrupting the pathways of IRI [8]. The identification of effective pharmacological agents could expand the available options for surgeons and allow for the use of liver grafts with EDC for transplantation. Unfortunately, promising agents and strategies against IRI have not become part of the clinical routine yet. Additionally, there are few systematically summarized reports which are limited in rat animal model experiments as preclinical studies.

The aim of this study is to systematically review the reported literature in which pharmacological agents against IRI have been studied using rat LT models. Additionally, the study is focused on finding pharmaceutical strategies that could be used in clinical routine as a mean of categorizing the identified studies according to the pathology of hepatic IRI.

## Materials and Methods

### Literature search

A systematic search of the PubMed database for literature reported in the period between January 1993 and December 2012 was performed. The search parameters were restricted to studies reported in the English language that had an available online abstract. The search command used for the review was “(rat liver transplantation) AND (preconditioning OR pharmacological OR drug OR modification) NOT (partial) NOT (small for size) NOT (ischemic preconditioning)”. In addition, literature that examined the identified agents as clinical trial candidates were also assessed for future clinical application. All experimental studies to examine pharmacological agents that were effective against IRI by means of rat LT models were included. Studies were excluded if one or more of the following conditions were applicable: 1) rat models in which machine perfusion, isolated perfused liver, *ex vivo* treatment, *ex vivo* perfusion, xenograft, or partial LT procedures were performed, 2) non-heart beating donors, brain dead models, or fatty liver models, 3) the presence of gene transfection or potentially harmful agents, and 4) a pharmacological agent that was principally used as an immunosuppressant. This systematic review was examined according to PRISMA guideline [9].

### Included Studies

**Fig 1:**
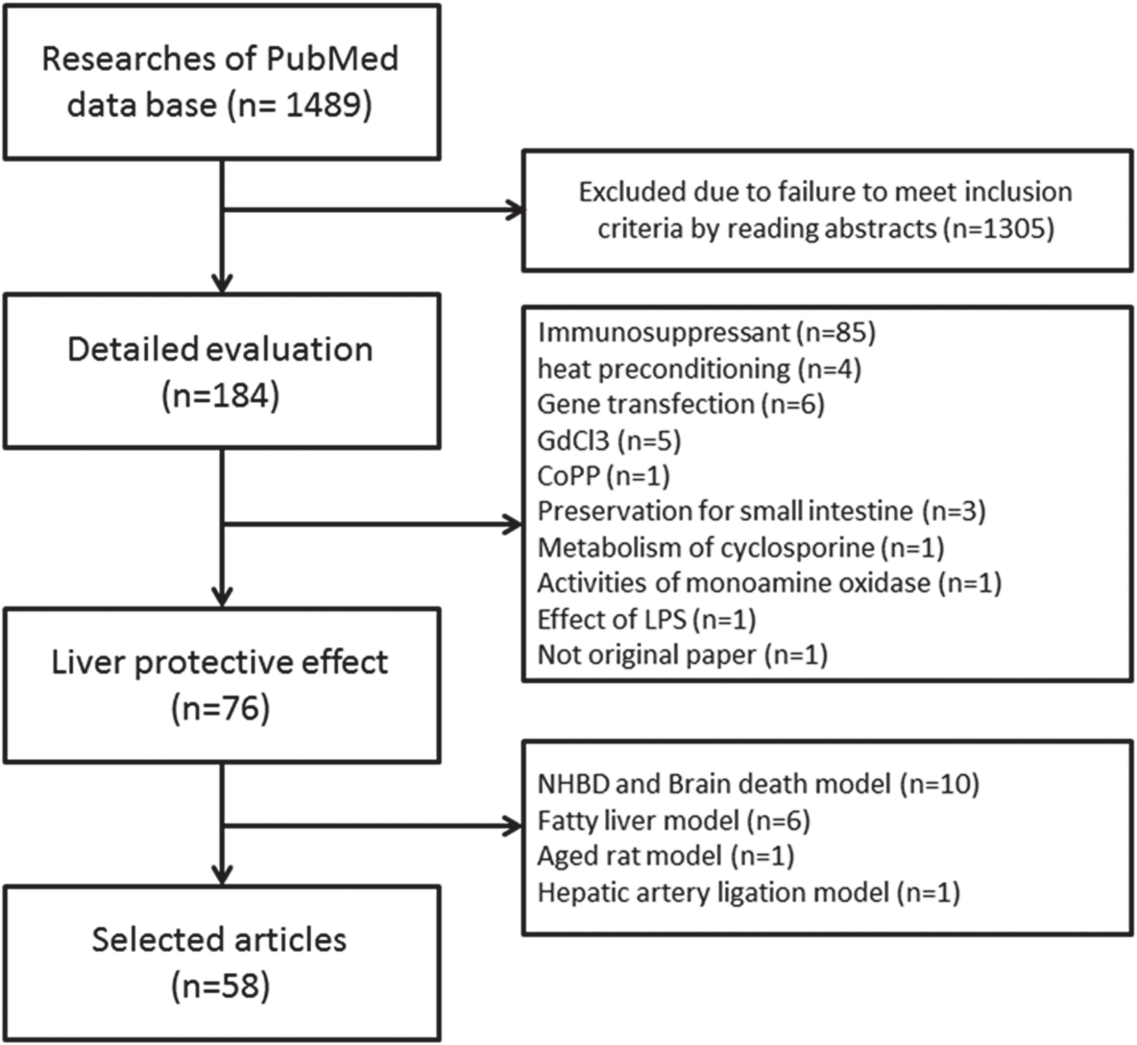
Study flow diagram included in the systematic review.

The database search yielded 1489 studies, of which 184 studies reported the effects of pharmacological agents on rat LT models. In the end, a total of 58 articles could be included in this review (Fig 1) [10–67].

### Data extraction and outcome measures

Data on the type of rat models used in each study, the species and number of rats in the model, the type of cold preservation solution, the cold ischemia time (CIT), hepatic artery reconstruction (HAR), and donor and/or recipient treatment protocols were extracted from the articles. The 7-day survival rates were used to perform a meta-analysis. [68] Approximately 2700 rats underwent LT. All studies used syngeneic rat LT models. In thirty studies, HAR was performed. Pharmacological agents were administered as donor- and/or recipient-treated regimens; 29 studies examined the effect of donor preconditioning, 21 studies focused on recipient treatment options and 8 studies looked at a combined donor-recipient treatment option. The subsequent survival benefit was examined in 31 studies. Transaminases were detected with several methods at various timepoints after LT; thus, these parameters were not compared to assess the effects of an agent.

### Categorization of pharmacological agents according to the pathology of the hepatic IRI

The pharmaceutical agents were categorized as follows: І—adenosine and nitric oxide (NO) agonists, endothelin (ET) antagonists, and prostaglandins (PGs), II—KC inactivators, III—complement inhibiters, IV—antioxidants, V—neutrophil inactivators, VI—anti-apoptosis agents, VII—heat shock protein (HSP) and nuclear factor *kappa* B (NF-κB) inducers, VIIІ—metabolic agents, ІХ—agents used in traditional Chinese medicine, and Х—others (Table 1).

**Table 1:** Characteristics of experimental studies included in the systematic review.

Drug category (n = 58)	Author, Year	Treatment drug	Treatment	Species	Number	Solution	CI time	HAR	Survival study
Group I Adenosine agonist, NO agonist, ET antagonist, PGs (n = 13)	Ghonem N, 2011^10^	Treprostinil	D	Lewis	50	UW	18h	No	No
	Oshima K, 2009^11^	FK3311	R	Lewis	71	UW	18h	No	Yes
	Huser N, 2009^12^	FK506, Aminoguanidine	D	DA	41	-	2h	Yes	No
	Farmer DG, 2008^13^	Tezosenta	R	SD	28	UW	24h	No	Yes
	Reid KM, 2007^14^	nor-NOA	R	Lewis	36	UW	18h	No	No
	Tsuchihashi S, 2006^15^	FK330	D,R	SD	48	UW	30, 40, 48h	No	Yes
	Yangnik GP, 2002^16^	L-arginine	R	Lewis	48	UW	18h	No	No
	Geller DA, 2001^17^	L-arginine	R	Lewis	-	UW	18h	No	No
	Tian YH, 2000^18^	Adenosine deaminase inhibitor	D	Lewis	23	UW	44h	No	Yes
	Tanaka W, 2000^19^	TAK-044	D	Wistar	60	EC	1h	No	No
	Liu H, 1998^20^	Prostaglandin E1	D	Wistar	16	EC	6h	No	No
	Xu HS, 1994^21^	Prostaglandin E1	R	SD	97	NS		No	Yes
	Maeda T, 1998^22^	cAMP, cGMP	D,R	Lewis	112	UW	24h	No	Yes
Group II KC inactivator (n = 10)	Sun K, 2012^23^	Taurine	R	SD	64	UW	1h	No	Yes
	Schemmer P, 2005^24^	Taurine	D	SD	86	HTK	4h	Yes	Yes
	Kamo N, 2011^25^	Sotraustaurin	D,R	SD	38	UW	30h	Yes	Yes
	Chimalakonda AP, 2007^26^	Methylpredonisole (DMP)	D	SD	45	UW	24h	No	Yes
	Liu ZJ, 2006^27^	Glycine	D	SD	80	UW	1h	No	Yes
	Rentsch M, 2005^28^	Glycine	D	Lewis	69	UW	24h	Yes	Yes
	Schemmer P, 1999^29^	Glycine	D	Lewis	54	UW	1h	Yes	Yes
	Urata K, 2000^30^	Nisoldipine, Thalidomide	D	Lewis	24	UW	24h	No	Yes
	Hashimoto K, 2000^31^	FR167653	R	BN	36	UW	48h	No	Yes
	Nishizawa H, 1997^32^	Pentoxyfylline	D	Lewis	36	UW	12h	No	No
Group III Complement inhibiter (n = 2)	Zhang J, 2011^33^	Complement with CV factor	D	Wistar	12	UW	2h	No	No
	Lehmann TG, 1998^34^	Soluble complement receptor 1	R	Lewis	16	UW	24h	Yes	No
Group IV Antioxidant (n = 4)	Schauer RJ, 2004^35^	Glutathione	R	Lewis	36	UW	24h	Yes	No
	Koeppel TA, 1996^36^	N-acetylcycteine	D,R	Lewis	16	UW	24h	Yes	No
	Walcher F, 1995^37^	N-acetylcycteine	D,R	SD	12	UW	20h	No	No
	Consenza CA, 1994^38^	Lazaroid U74006F	D	Lewis	30	UW	24h	No	Yes
Group V Neutrophil inactivator (n = 6)	Schen XD, 2007^39^	Diannexin	R	SD	61	UW	24h	No	Yes
	Tsuchihashi S, 2006^40^	anti-PSGL	R	Lewis	32	UW	24h	No	Yes
	Soejima Y, 1999^41^	ONO-5046	R	Lewis	24	Ringer	5h	No	No
	Dulkanchainun TS, 1998^42^	sPSGL-1	D,R	SD	20	UW	24h	No	Yes
	Anthuber M, 1997^43^	Enalapril	R	Lewis	18	UW	24h	Yes	No
	Walcher F, 1996^44^	WEB2086	R	SPRD	26	UW	5h	No	No
Group VI Anti-apoptosis agent (n = 4)	Grutzner U, 2006^45^	ANP	D	Lewis	16	UW	24h	Yes	No
	Nowak G, 2005^46^	UDCA	D	Wistar	12	UW	8h	Yes	No
	Meuller TH, 2004^47^	DEVD-fluoromethylketone	D	Lewis	54	UW	16h	Yes	Yes
	Natori S, 1999^48^	IDN-1965	D,R	Lewis	10	UW	30h	No	Yes
Group VII HSP, NFκB inducer (n = 7)	Zeng Z, 2012^49^	Diazoxide	D	SD	80	UW	4h	No	No
	Cheng MX, 2012^50^	NBD peptides	D	SD	48	UW	18h	No	No
	Kaizu T, 2008^51^	Carbon monoxide	R	Lewis	42	UW	18h	No	No
	Fondevila C, 2004^52^	Biliverdin	D,R	SD	152	UW	24h	No	Yes
	Tsuchihashi S, 2003^53^	Pyrrolidine dithiocarbamate	D	Lewis	47	UW	24h	No	Yes
	Fudaba Y, 2001^54^	Geranylgeranylacetone	D	BN	46	NS	45min*	No	Yes
	Fudaba Y, 2000^55^	Geranylgeranylacetone	D	BN	20	NS	45min*	No	Yes
Group VIII Metabolic agent (n = 2)	Ma ZW, 2007^56^	Fat emulsion	R	SD	96	Ringer	15min	No	Yes
	Morimoto Y, 1996^57^	Insulin	D	Lewis	28	UW	24h	No	No
Group IX Traditional Chinese medicine (n = 6)	Song S 2010^58^	Sinomenine	D	SD	76	UW	24h	No	Yes
	Liang R, 2009^59^	Danshen	D	SD	52	Ringer	1h	Yes	Yes
	Chen T, 2012^60^	Shenfu	R	SD	96	-	100min	No	No
	Zhu WH, 2006^61^	Shenfu	R	SD	30	NS	4h	No	No
	Zhu X, 2003^62^	Matrine	D	SD	80	Ringer	5h	No	Yes
	Zhu XH, 2003^63^	Matrine	D	SD	72	Ringer	5h	No	No
Group X Others (n = 4)	Tarrab E, 2012^64^	Cyclosporin-A	D	Lewis	17	UW	24h	No	No
	Chen LP, 2010^65^	Rapamycin	R	Wistar	128	UW	12h	Yes	No
	Gao W, 1997^66^	Minocycline, IFNα, Fumagillin	D	Lewis	14	EC	16h	Yes	Yes
	Terakura M, 1995^67^	Putrescine	R	Wistar	16	EC	6h	No	No

NO: nitric oxide, ET: endothelin, PGs: prostaglandins, KC: Kupffer cell, NFκB: nuclear factor *kappa* B, CV: cobra venom, INF: interferon, HAR: hepatic artery reconstruction, SD: Sprague-Dawley, BN: Brown Norway, DA: Dark Agouti, D: Donor, R: Recipient, EC: Euro–Collins solution, UW: University of Wisconsin solution, NS: normal saline, UDCA: ursodeoxycholic acid,

*: 37°C, -:not estimated

Group I agents were known to generally preserve microvascular structure and microcirculation in the liver. Treprostinil, a PGI_2_ analog, plays a critical role in microcirculation [10], and the selective COX-2 inhibitor, FK3311, prevents platelet aggregation and causes vasodilatation [11]. Enalapril is a ACE inhibitor that acts by inducing vasodilation via different pathways [43].

Sotraustaurin is an immunosuppressant that prevents early T-cell activation via a calcineurin-independent pathway. Sotraustaurin treatment was reported to be linked with T-cell-macrophage crosstalk [24]. FR167653 is a potent suppressant of IL-1β and TNF-α production in monocytes and has been reported to be associated with the reduced expression of TF in KCs [31]. It is for these reasons that sotraustaurin and FR167653 were categorized in Group ІІ.

### Statistical Analysis

Both the Risk Ratio (RR) and the 95% confidence Interval (CI) for the 7-day survival probability were determined using Mantel-Haenszel´s Random Effects model. The *I* ^2^ statistics were calculated in order to assess the heterogeneity of the studies under review. The *I* ^2^ values of 0%, 25%, 50% and 75% were estimated as “No”, “Low”, “Moderate” and “High” heterogeneity, respectively [69]. A two-tailed *p* value of less than 0.05 was deemed statistically significant. All statistical analyses were performed using Review Manager, Version 5 (The Cochrane Collaboration, Oxford, UK).

## Results

### Agents that deactivated Kupffer cells and agents that induced HSP and NF-κB were mostly used for donor preconditioning, whereas the agents that prohibited neutrophil activation were administered during recipient treatment

**Fig 2:**
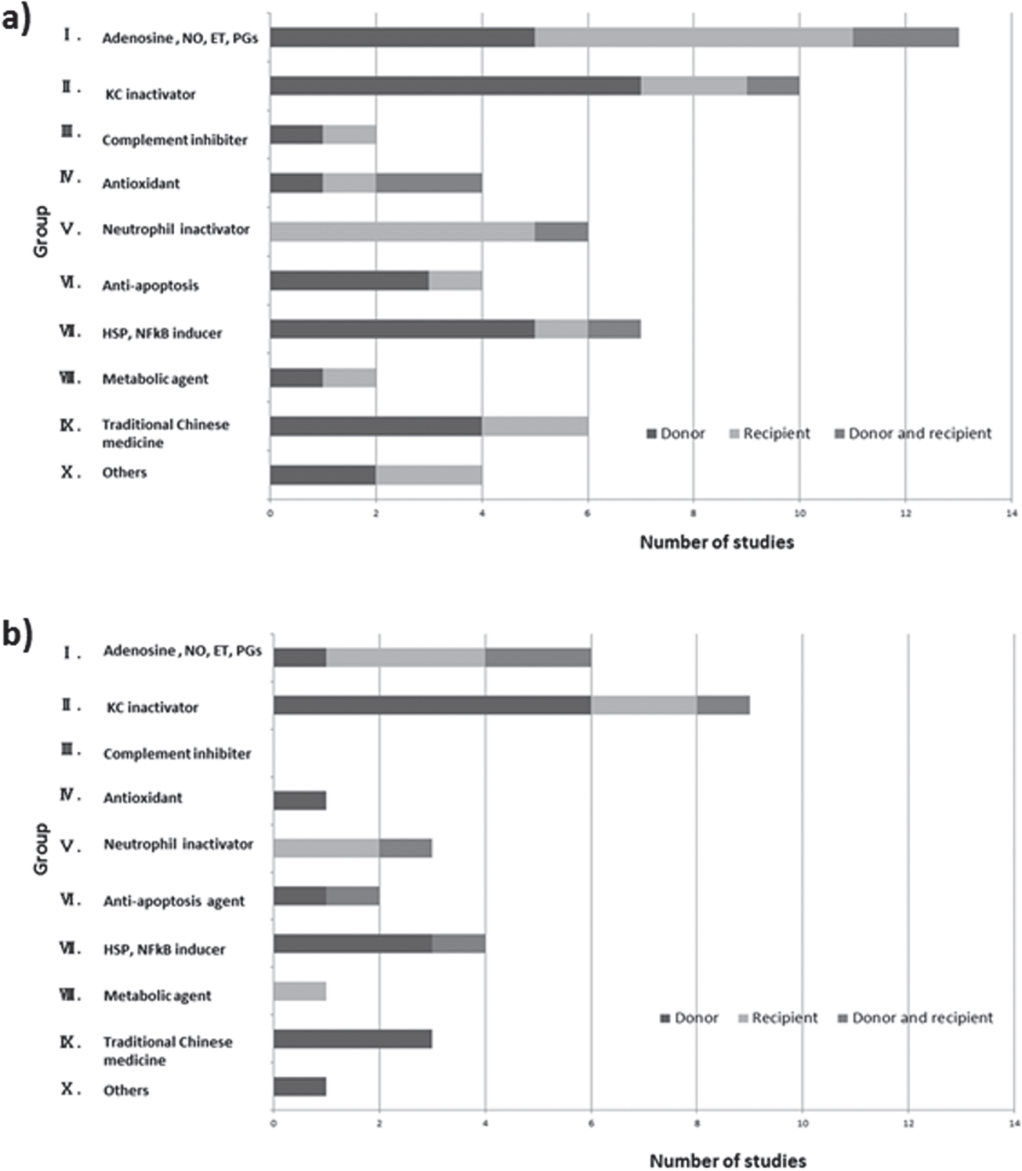
Categorization and number of studies in total (a) and in the subgroup analysis that examined survival benefits (b).

The number of studies focused on each type of pharmaceutical agent was: Group І- 13 studies, Group II—10 studies, Group III—2 studies, Group IV—4 studies, Group V—6 studies, Group VI—4 studies, Group VII—7 studies, Group VIII—2 studies, Group ІХ- 6 studies, Group Х- 4 studies in total. The number of donor-treated experiments, recipient-treated experiments and both treated experiments and the rate in each group was 5 (39%), 6 (46%), 2 (15%) in Group І, 7 (70%), 2 (20%), 1 (10%) in Group II, 1 (50%), 1 (50%), 0 (0%) in Group III, 1 (25%), 1 (25%), 2 (50%) in Group IV, 0 (0%), 5 (83%), 1 (16%) in Group V, 3 (75%), 1 (25%), 0 (0%) in Group VI, 5 (71%), 1 (14%),1 (14%) in Group VII, 1 (50%), 1 (50%), 0 (0%) in Group VIII, 4 (67%), 2 (33%), 0 (0%) in Group ІХ, 2 (50%), 2 (50%), 0 (0%) in Group Х, respectively (Fig 2A). The differences of the rates of donor and/or recipient were observed among the 10 groups, suggesting that the categorization might predict suitable phase of treatment options. Most notably, the rates of donor-treated experiment were highest in group ІІ (70%) and VII (71%), whereas the rate in recipient-treated experiments was higher in category V (83%).

### The agents that deactivated Kupffer cell potentially have short-term survival benefits

Of the 31 studies that examined survival benefit, only one was excluded from the subgroup analysis on the grounds that it did not use a control group.^55^ The number of the studies that examined survival benefit in each group is as follows: Group І- 6 studies, Group II—9 studies, Group III—0 studies, Group IV—1 study, Group V—3 studies, Group VI—2 studies, Group VII—4 studies, Group VIII—1 study, Group ІХ- 3 studies, group Х- 1 study (Fig 2B). The number of the studies in Group I decreased from thirteen to six. Meanwhile, the number in group II decreased only from ten to nine, giving impression that agents in Group II were more likely to offer short-term survival benefits. In the subgroup analysis, the number and rates of experimental studies in donor-treated experiments, recipient-treated experiments, and both donor and recipient-treated experiments were 1 (17%), 3 (50%), 2 (33%) in Group І, 6 (67%), 2 (22%), 1 (11%) in Group ІІ, 0 (0%), 2 (67%), 1 (33%) in Group V, 3 (75%), 0 (14%),1 (25%) in Group VII, and 3 (100%), 0 (0%), 0 (0%) in Group ІХ, respectively (Fig 2B). The rates of donor-treated experiments in group ІІ and VII were 67% and 75%, and that of recipient-treated experiments in group V was 67%. In Group I, however, the rate of the number of donor-treated experiments decreased from 39% to 17%, suggesting that agents in Group I provide relatively less short-term survival benefits.

### The meta-analysis demonstrated that the Risk Ratio was 2.43 [95% CI: 1.88–3.14] with moderate heterogeneity

**Fig 3:**
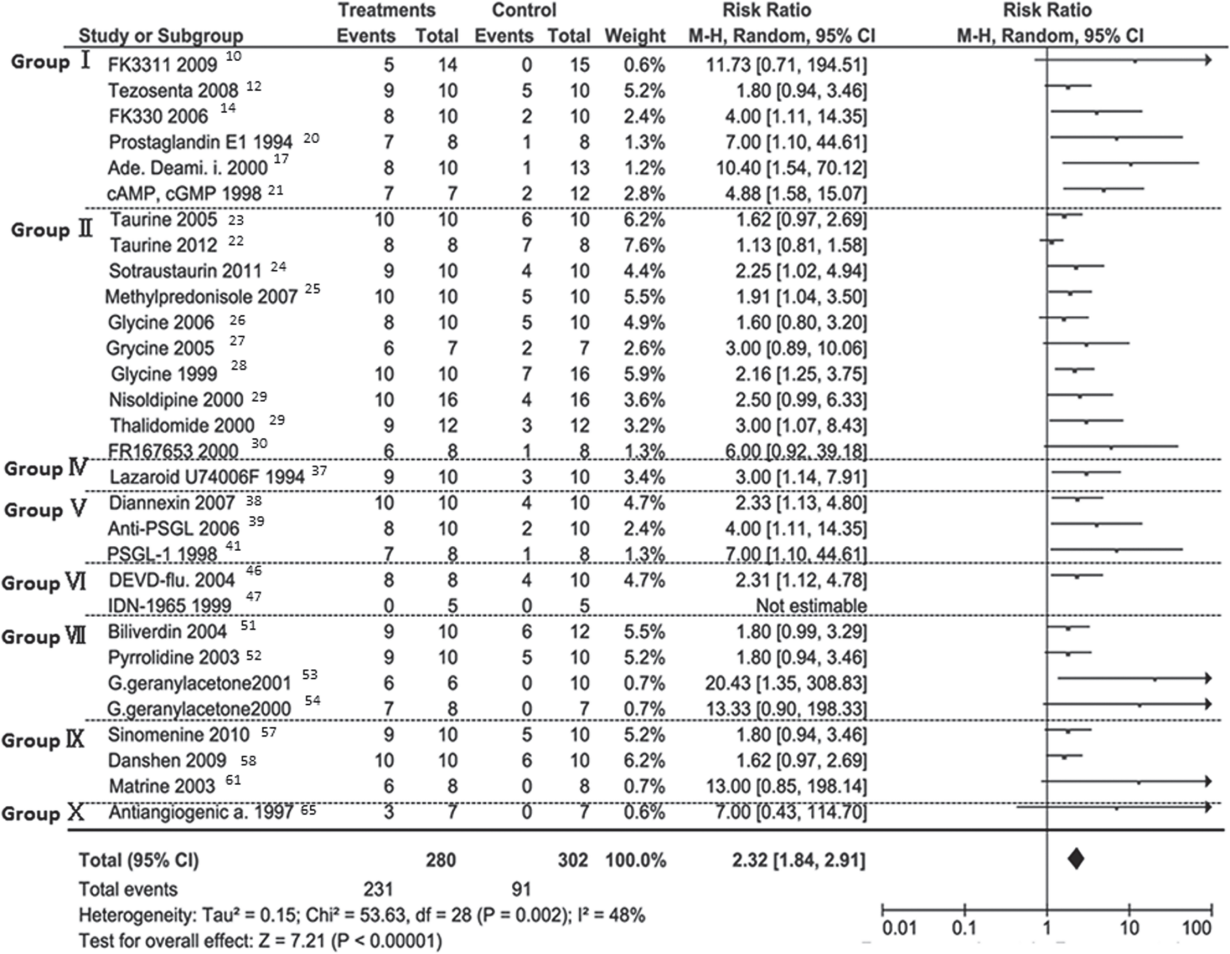
Annotated forest plot for meta-analysis of risk ratio of seven-day-survival probability.

The meta-analysis showed that RR was 2.43 [95% CI: 1.88–3.14] (Fig 3). However, moderate heterogeneity was observed with statistical significance (*I* ^2^ = 48%, P = 0.002). In the subgroup analysis in which experimental conditions of 24 hours CIT with University of Wisconsin (UW) solution were used (n = 13), RR was 2.21 [95% CI: 1.77–2.75] and no heterogeneity was observed (*I* ^2^ = 0, P = 0.87). In addition, if the subgroup was divide into donor- and/or recipient-treatment regimens, the RR obtained for donor-treated experiment was 2.49 [95% CI: 1.78–3.50], for the recipient-treated experiment was 2.20 [95% CI: 1.40–3.47], and for the both-treated experiment was 2.14 [1.28–3.55], respectively.

## Discussion

This is the first systematic review and meta-analysis of the efficacy of pharmacological agents in rat LT models. The result of meta-analysis using the 7-day survival rate showed that pharmacological agents conferred short-term survival benefits that were probably associated with the prevention of PNF and PDF. Pharmacological treatment is believed to be effective to reduce IRI in LT, because their benefits in survival after LT have been proven by experimental researches. Therefore, based on the experimental data that are available today, the identified agents should be further evaluated in human LT. Actually, among the identified agents, methylprednisolone, a pan-caspase inhibitor, recombinant P-selectin glycoprotein ligand (rPSGL-Ig), and N-acetylcysteine (NAC) have already been studied in clinical trials. The agents except NAC have short-term survival benefits that are proven by the identified experimental researches. However, none of the pharmacological agents against IRI have become part of the clinical routine.

First of all, we would consider the results of the reported clinical trials to clarify why the pharmacological agents against IRI in LT are not established as the clinical routine. One study on the effects of methylprednisolone revealed that the administration of the agent reduced the levels of cytokines in donor subjects and preserved the graft function (which was estimated by examining the aspartate aminotransferase (AST) and alanine aminotransferase (ALT) levels [70], whereas another research group showed that methylprednisolone treatment conferred little to no survival benefits and was associated with a higher risk oh biopsy-confirmed rejection [71]. Baskin-Bey, *et al*. reported that when a pan-caspase inhibitor was administered only to storage and flash solutions, it reduced the prevalence of graft injury. However, treating the recipient with this agent had detrimental consequences [72], even if the pan-caspase inhibitor administered in the trial under investigation was IDN-6556 and not the variant IDN-1965. RPSGL-Ig was used for recipient-treated procedures, as well as in *ex vivo* liver flushes [73]. In patients with a donor risk index above the accepted study average, administration of rPSGL-Ig improved serum AST levels. Weigand´s study on the effectiveness of NAC revealed that the agent inhibited the increase in glutathione S-transferase (αGST), serum intercellular adhesion molecule (ICAM)-1, and vascular cell adhesion molecule (VCAM)-1 levels after reperfusion of the donor liver [74]. However, Hilmi, *et al*. reported that NAC was ineffective against reducing the risk of acute kidney injury after LT and was not beneficial in terms of liver function or subject survival [75]. None of these agents resulted in a decrease in the mortality rate, liver failure, or perioperative morbidity in clinical setting, even though some promising pharmaceuticals engendered an improvement in the secondary outcomes of AST, ALT, and some other molecules. Thus, none of these agents resulted in a decrease in the mortality rate, liver failure, or perioperative morbidity in clinical trials. From this point of view, this study revealed other promising agents that had beneficial effects against IRI in LT as shown in Table 1. However, the differences in the RR among identified studies were too model-dependent to be used to find out the most promising agent because each experiment used different cold preserve solutions and CIT with or without HAR. Considering the fact that none of these agents decrease the mortality rate in clinical setting, obtaining the RR of only 2.5 times in the meta-analysis (which could be achieved by each single agent) might be too small to achieve definitive effects against hepatic IRI. Additionally, there are relatively small differences in the observed RR among the donor- and/or recipient- treated subgroups, suggesting that it is unclear which phase is more critical for pharmacological treatment. Therefore, additional strategies will need to be investigated in order to find an action plan that will effectively overcome the complexity of IRI in the clinical setting. Since a rat liver transplantation model is technical demanding, there is only a limited number of publications in contrast to studies using IRI to mimic in part what occur after liver transplantation. A transplant model is a more clinically relevant and thus should be used to address the question whether a new agent may be beneficial to prevent livers from IRI in LT. To increase the number of the studies that can be analyzed large animal studies were included; the effects of ET receptor antagonist (TAK-004) [76], L-arginine [77] and N-acetylcysteine [78] were proved by a pig liver transplantation model as well as a rat liver transplantation model. Four agents which were not included in Table 1 were found, a selective ET_A_ receptor antagonist (BSF208075) [79], thromboxane A_2_ synthase inhibitor (sodium ozagrel) [80], platelet-activating factor antagonist (E5880) [81], Cardiotrophin-1, which is a cytokine belonging to the IL-6 family [82]**.**

Multifactorial and pleiotropic approaches have been advocated for simultaneous action on several IRI pathologies [9, 83]. However, very few studies have reported on the effectiveness of cocktail treatments as potential pharmacological strategies for clinical application [84]. From the result of this review, the agents that deactivate KCs and the agents that induce HSP and NF-κB can be used in donor preconditioning and the agents that prohibit neutrophil activation can be administered in recipient courses. Additionally, it has been determined that agents classified as KC inactivators can be administered with the aim of engendering short-term benefits after reperfusion. Thus, multifactorial and pleiotropic approaches based on the stated categorizations could be designed as a first step with the pharmacological effects in donor and/or recipient treatment being taken into full consideration. In our manuscript, all the agents were categorized based on the findings of the evaluated publications.

Secondly, the degree of IRI is dependent on the length and method of ischemia applied to the liver as well as the background condition of the organ [85]. For example, liver steatosis is an important risk factor for IRI in the clinical setting [86]. Differences in the action mechanisms that occur in steatotic and non-steatotic livers were observed [87]. The following drugs were reportedly examined in several studies using fatty liver models that were excluded from this review: a cyclin RGD peptide, recombinant human erythropoietin, and fibronectin-α4β1 integrin [88–91]. Due to the fact that these agents were not used in non-steatotic models, they have not been included in the selected literatures of this review. Therefore, pharmacological effects of a newly designed multifactorial and pleiotropic approach could be examined using different background liver conditions.

Finally, the additional or synergic effects, in combination with the different categories of agents to be used in multifactorial and pleiotropic approaches, should be examined. It must be noted that it would be extremely difficult to anticipate and measure these effects without a biomarker, which could be integrated into the complex pathology of hepatic IRI. Several studies regarding Damage-associated Molecular Patterns (DAMPs) in hepatic IRI were recently published. DAMPs are interestingly indicators of tissue injury as well as first line responders of immunological systems in LT [85, 92], as such, they might be useful biomarkers when examining short- and/or long-term survival benefits of multifactorial and pleiotropic treatment. Biomarkers including AST and ALT should be investigated in a parallel manner in order to measure pharmacological effects and to establish multifactorial and pleiotropic approaches in experimental LT models.

In conclusion, pharmacological strategies could be effective in reducing IRI in LT. The agents identified in this study should be further evaluated in human LT. However, further development of the strategies will be needed in order to better determine the effectiveness of agents in clinical application. The categorization of agents with consideration to hepatic IRI pathology might be the first step in designing multifactorial and pleiotropic approaches in rat LT models.

## Supporting Information

S1 PRISMA Checklist Meta-analysis on Genetic Association Studies Checklist. (DOCX)

## References

[1] Clavien PA, Lesurtel M, Bossuyt PM, Gores GJ, Langer B, Perrier A. Recommendations for liver transplantation for hepatocellular carcinoma: an international consensus conference report. Lancet Oncol. 2012; 13:e11–22. 10.1016/S1470-2045(11)70175-9 22047762 PMC3417764

[2] Carithers RL Jr. Liver transplantation. American Association for the Study of Liver Diseases. Liver Transpl. 2000; 6:122–135. 10648593 10.1002/lt.500060122

[3] Johnson SR, Alexopoulos S, Curry M, Hanto DW. Primary nonfunction (PNF) in the MELD Era: An SRTR database analysis. Am J Transplant. 2007; 7:1003–1009. 17286618 10.1111/j.1600-6143.2006.01702.x

[4] Malhi H, Gores GJ, Lemasters JJ. Apoptosis and necrosis in the liver: a tale of two deaths? Hepatology. 2006; 43:S31–44. 16447272 10.1002/hep.21062

[5] Busuttil RW, Tanaka K. The utility of marginal donors in liver transplantation. Liver Transpl. 2003; 9:651–663. 12827549 10.1053/jlts.2003.50105

[6] Weigand K, Brost S, Steinebrunner N, Buchler M, Schemmer P, Muller M. Ischemia/Reperfusion injury in liver surgery and transplantation: pathophysiology. HPB Surg. 2012; 2012:176723 10.1155/2012/176723 22693364 PMC3369424

[7] Selzner N, Rudiger H, Graf R, Clavien PA. Protective strategies against ischemic injury of the liver. Gastroenterology. 2003; 125:917–936. 12949736 10.1016/s0016-5085(03)01048-5

[8] de Rougemont O, Dutkowski P, Clavien PA. Biological modulation of liver ischemia-reperfusion injury. Curr Opin Organ Transplant. 2010; 15:183–189. 10.1097/MOT.0b013e3283373ced 20125019

[9] Moher D, Liberati A, Tetzalff J, Altman DG, The PRISMA Group. Preferred Reporting Items for Systematic Reviews and Meta-Analyes: The PRISMA Statement. PLOS Med. 2009; 6: e1000097 10.1371/journal.pmed.1000097 19621072 PMC2707599

[10] Ghonem N, Yoshida J, Stolz DB, Humar A, Starzl TE, Murase N, et al Treprostinil, a prostacyclin analog, ameliorates ischemia-reperfusion injury in rat orthotopic liver transplantation. Am J Transplant. 2011; 11:2508–2516. 10.1111/j.1600-6143.2011.03568.x 21668631

[11] Oshima K, Yabata Y, Yoshinari D, Takeyoshi I. The effects of cyclooxygenase (COX)-2 inhibition on ischemia-reperfusion injury in liver transplantation. J Invest Surg. 2009; 22:239–245. 19842898 10.1080/08941930903040080

[12] Huser N, Doll D, Altomonte J, Werner M, Kriner M, Preissel A, et al Graft preconditioning with low-dose tacrolimus (FK506) and nitric oxide inhibitor aminoguanidine (AGH) reduces ischemia/reperfusion injury after liver transplantation in the rat. Arch Pharm Res. 2009; 32:215–220. 10.1007/s12272-009-1138-9 19280151

[13] Farmer DG, Kaldas F, Anselmo D, Katori M, Shen XD, Lassman C, et al Tezosentan, a novel endothelin receptor antagonist, markedly reduces rat hepatic ischemia and reperfusion injury in three different models. Liver Transpl. 2008; 14:1737–1744. 10.1002/lt.21621 19025917 PMC2975480

[14] Reid KM, Tsung A, Kaizu T, Jeyabalan G, Ikeda A, Shao L, et al Liver I/R injury is improved by the arginase inhibitor, N(omega)-hydroxy-nor-L-arginine (nor-NOHA). Am J Physiol Gastrointest Liver Physiol. 2007; 292:G512–517. 17023552 10.1152/ajpgi.00227.2006

[15] Tsuchihashi S, Kaldas F, Chida N, Sudo Y, Tamura K, Zhai Y, et al FK330, a novel inducible nitric oxide synthase inhibitor, prevents ischemia and reperfusion injury in rat liver transplantation. Am J Transplant. 2006; 6:2013–2022. 16796718 10.1111/j.1600-6143.2006.01435.x

[16] Yagnik GP, Takahashi Y, Tsoulfas G, Reid K, Murase N, Geller DA. Blockade of the L-arginine/NO synthase pathway worsens hepatic apoptosis and liver transplant preservation injury. Hepatology. 2002; 36:573–581. 12198649 10.1053/jhep.2002.35058

[17] Geller DA, Chia SH, Takahashi Y, Yagnik GP, Tsoulfas G, Murase N. Protective role of the L-arginine-nitric oxide synthase pathway on preservation injury after rat liver transplantation. JPEN J Parenter Enteral Nutr. 2001; 25:142–147. 11334063 10.1177/0148607101025003142

[18] Tian YH, Schafer T, Sckell A, Schilling MK. Adenosine deaminase inhibition attenuates reperfusion low flow and improves graft survival after rat liver transplantation. Transplantation. 2000; 69:2277–2281. 10868626 10.1097/00007890-200006150-00010

[19] Tanaka W, Yamanaka N, Onishi M, Ko M, Yamanaka J, Okamoto E. Optimal route of administration of mixed endothelin receptor antagonist (TAK-044) in liver transplantation. J Gastroenterol. 2000; 35:120–126. 10680667 10.1007/s005350050024

[20] Liu H, Wang L, Liu Y, Song J, Huang J, He S. Experimental study on liver microcirculation disturbance following transplantation and the protective effect of prostaglandin E1 in the rat. Chin Med J (Engl). 1998; 111:1079–1082. 11263368

[21] Terakura M, Higaki I, Matsui-Yuasa I, Kinoshita H, Otani S. Polyamine metabolism in the rat liver after orthotopic liver transplantation. Biochim Biophys Acta. 1995; 1245:207–214. 7492579 10.1016/0304-4165(95)00100-p

[22] Maeda T, Murase N, Subbotin V, Sakamoto T, Yamada T, Terakura M, et al Analogs of cyclic nucleotides in rat liver preservation. Transplantation. 1998; 66:844–851. 9798692 10.1097/00007890-199810150-00006

[23] Sun K, Chen Y, Liang SY, Liu ZJ, Liao WY, Ou ZB, et al Effect of taurine on IRAK4 and NF-kappa B in Kupffer cells from rat liver grafts after ischemia-reperfusion injury. Am J Surg. 2012; 204:389–395. 10.1016/j.amjsurg.2011.10.020 22771449

[24] Schemmer P, Liang R, Kincius M, Flechtenmacher C, Bunzendahl H, Gutt CN, et al Taurine improves graft survival after experimental liver transplantation. Liver Transpl. 2005; 11:950–959. 16035074 10.1002/lt.20501

[25] Kamo N, Shen XD, Ke B, Busuttil RW, Kupiec-Weglinski JW. Sotrastaurin, a protein kinase C inhibitor, ameliorates ischemia and reperfusion injury in rat orthotopic liver transplantation. Am J Transplant. 2011; 11:2499–2507. 10.1111/j.1600-6143.2011.03700.x 21883905 PMC3625141

[26] Chimalakonda AP, Mehvar R. Effects of methylprednisolone and its liver-targeted dextran prodrug on ischemia-reperfusion injury in a rat liver transplantation model. Pharm Res. 2007; 24:2231–2238. 17922174 10.1007/s11095-007-9414-1 PMC2094048

[27] Liu ZJ, Yan LN, Li SW, You HB, Gong JP. Glycine blunts transplantative liver ischemia-reperfusion injury by downregulating interleukin 1 receptor associated kinase-4. Acta Pharmacol Sin. 2006; 27:1479–1486. 17049125 10.1111/j.1745-7254.2006.00413.x

[28] Rentsch M, Puellmann K, Sirek S, Iesalnieks I, Kienle K, Mueller T, et al Benefit of Kupffer cell modulation with glycine versus Kupffer cell depletion after liver transplantation in the rat: effects on postischemic reperfusion injury, apoptotic cell death graft regeneration and survival. Transpl Int. 2005; 18:1079–1089. 16101730 10.1111/j.1432-2277.2005.00185.x

[29] Schemmer P, Bradford BU, Rose ML, Bunzendahl H, Raleigh JA, Lemasters JJ, et al Intravenous glycine improves survival in rat liver transplantation. Am J Physiol. 1999; 276:G924–932. 10198336 10.1152/ajpgi.1999.276.4.G924

[30] Urata K, Brault A, Rocheleau B, Huet PM. Role of Kupffer cells in the survival after rat liver transplantation with long portal vein clamping times. Transpl Int. 2000; 13:420–427. 11140240 10.1007/s001470050724

[31] Hashimoto K, Nishizaki T, Yoshizumi T, Uchiyama H, Okano S, Ikegami T, et al Beneficial effect of FR167653 on cold ischemia/reperfusion injury in rat liver transplantation. Transplantation. 2000; 70:1318–1322. 11087146 10.1097/00007890-200011150-00009

[32] Nishizawa H, Egawa H, Inomata Y, Uemoto S, Asonuma K, Kiuchi T, et al Efficiency of pentoxifylline in donor pretreatment in rat liver transplantation. J Surg Res. 1997; 72:170–176. 9356239 10.1006/jsre.1997.5169

[33] Zhang J, Hu W, Xing W, You T, Xu J, Qin X, et al The protective role of CD59 and pathogenic role of complement in hepatic ischemia and reperfusion injury. Am J Pathol. 2011; 179:2876–2884. 10.1016/j.ajpath.2011.08.040 22019898 PMC3260856

[34] Lehmann TG, Koeppel TA, Kirschfink M, Gebhard MM, Herfarth C, Otto G, et al Complement inhibition by soluble complement receptor type 1 improves microcirculation after rat liver transplantation. Transplantation. 1998; 66:717–722. 9771834 10.1097/00007890-199809270-00005

[35] Schauer RJ, Kalmuk S, Gerbes AL, Leiderer R, Meissner H, Schildberg FW, et al Intravenous administration of glutathione protects parenchymal and non-parenchymal liver cells against reperfusion injury following rat liver transplantation. World J Gastroenterol. 2004; 10:864–870. 15040034 10.3748/wjg.v10.i6.864 PMC4726997

[36] Koeppel TA, Lehmann TG, Thies JC, Gehrcke R, Gebhard MM, Herfarth C, et al Impact of N-acetylcysteine on the hepatic microcirculation after orthotopic liver transplantation. Transplantation. 1996; 61:1397–1402. 8629304 10.1097/00007890-199605150-00020

[37] Walcher F, Marzi I, Flecks U, Larsen R. N-acetylcysteine failed to improve early microcirculatory alterations of the rat liver after transplantation. Transpl Int. 1995; 8:317–323. 7546156 10.1007/BF00346887

[38] Cosenza CA, Cramer DV, Cunneen SA, Tuso PJ, Wang HK, Makowka L. Protective effect of the lazaroid U74006F in cold ischemia-reperfusion injury of the liver. Hepatology. 1994; 19:418–425. 8294099

[39] Shen XD, Ke B, Zhai Y, Tsuchihashi SI, Gao F, Duarte S, et al Diannexin, a novel annexin V homodimer, protects rat liver transplants against cold ischemia-reperfusion injury. Am J Transplant. 2007; 7:2463–2471. 17868064 10.1111/j.1600-6143.2007.01967.x

[40] Tsuchihashi S, Fondevila C, Shaw GD, Lorenz M, Marquette K, Benard S, et al Molecular characterization of rat leukocyte P-selectin glycoprotein ligand-1 and effect of its blockade: protection from ischemia-reperfusion injury in liver transplantation. J Immunol. 2006; 176:616–624. 16365457 10.4049/jimmunol.176.1.616

[41] Soejima Y, Yanaga K, Nishizaki T, Yoshizumi T, Uchiyama H, Sugimachi K. Effect of specific neutrophil elastase inhibitor on ischemia/reperfusion injury in rat liver transplantation. J Surg Res. 1999; 86:150–154. 10452882 10.1006/jsre.1999.5661

[42] Dulkanchainun TS, Goss JA, Imagawa DK, Shaw GD, Anselmo DM, Kaldas F, et al Reduction of hepatic ischemia/reperfusion injury by a soluble P-selectin glycoprotein ligand-1. Ann Surg. 1998; 227:832–840. 9637546 10.1097/00000658-199806000-00006 PMC1191386

[43] Anthuber M, Farkas S, Rihl M, Menger MD, Schildberg FW, Jauch KW, et al Angiotensin-converting enzyme inhibition by enalapril: a novel approach to reduce ischemia/reperfusion damage after experimental liver transplantation. Hepatology. 1997; 25:648–651. 9049213 10.1002/hep.510250326

[44] Walcher F, Marzi I, Fischer R, Bauer M, Larsen R. Platelet-activating factor is involved in the regulation of pathological leukocyte adhesion after liver transplantation. J Surg Res. 1996; 61:244–249. 8769973 10.1006/jsre.1996.0111

[45] Grutzner U, Keller M, Bach M, Kiemer AK, Meissner H, Bilzer M, et al PI 3-kinase pathway is responsible for antiapoptotic effects of atrial natriuretic peptide in rat liver transplantation. World J Gastroenterol. 2006; 12:1049–1055. 16534845 10.3748/wjg.v12.i7.1049 PMC4087896

[46] Nowak G, Noren UG, Wernerson A, Marschall HU, Moller L, Ericzon BG. Enteral donor pre-treatment with ursodeoxycholic acid protects the liver against ischaemia-reperfusion injury in rats. Transpl Int. 2005; 17:804–809. 15815896 10.1007/s00147-004-0703-x

[47] Mueller TH, Kienle K, Beham A, Geissler EK, Jauch KW, Rentsch M. Caspase 3 inhibition improves survival and reduces early graft injury after ischemia and reperfusion in rat liver transplantation. Transplantation. 2004; 78:1267–1273. 15548962 10.1097/01.tp.0000141095.06273.10

[48] Natori S, Selzner M, Valentino KL, Fritz LC, Srinivasan A, Clavien PA, et al Apoptosis of sinusoidal endothelial cells occurs during liver preservation injury by a caspase-dependent mechanism. Transplantation. 1999; 68:89–96. 10428274 10.1097/00007890-199907150-00018

[49] Zeng Z, Huang HF, He F, Wu LX, Lin J, Chen MQ. Diazoxide attenuates ischemia/reperfusion injury via upregulation of heme oxygenase-1 after liver transplantation in rats. World J Gastroenterol. 2012; 18:1765–1772. 10.3748/wjg.v18.i15.1765 22553400 PMC3332289

[50] Cheng MX, Gong JP, Chen Y, Liu ZJ, Tu B, Liu CA. NBD peptides protect against ischemia reperfusion after orthotopic liver transplantation in rats. J Surg Res. 2012; 176:666–671. 10.1016/j.jss.2011.12.005 22381173

[51] Kaizu T, Ikeda A, Nakao A, Tsung A, Toyokawa H, Ueki S, et al Protection of transplant-induced hepatic ischemia/reperfusion injury with carbon monoxide via MEK/ERK1/2 pathway downregulation. Am J Physiol Gastrointest Liver Physiol. 2008; 294:G236–244. 18006605 10.1152/ajpgi.00144.2007

[52] Fondevila C, Shen XD, Tsuchiyashi S, Yamashita K, Csizmadia E, Lassman C, et al Biliverdin therapy protects rat livers from ischemia and reperfusion injury. Hepatology. 2004; 40:1333–1341. 15565657 10.1002/hep.20480

[53] Tsuchihashi S, Tamaki T, Tanaka M, Kawamura A, Kaizu T, Ikeda A, et al Pyrrolidine dithiocarbamate provides protection against hypothermic preservation and transplantation injury in the rat liver: the role of heme oxygenase-1. Surgery. 2003; 133:556–567. 12773984 10.1067/msy.2003.124

[54] Fudaba Y, Ohdan H, Tashiro H, Ito H, Fukuda Y, Dohi K, et al Geranylgeranylacetone, a heat shock protein inducer, prevents primary graft nonfunction in rat liver transplantation. Transplantation. 2001; 72:184–189. 11477336 10.1097/00007890-200107270-00003

[55] Fudaba Y, Tashiro H, Ohdan H, Miyata Y, Shibata S, Shintaku S, et al Efficacy of HSP72 induction in rat liver by orally administered geranylgeranylacetone. Transpl Int. 2000; 13 Suppl 1:S278–281. 11112012 10.1007/s001470050341

[56] Ma ZW, Liu LD, Li K, Zhang YJ, Dong JH. Improvement of graft function and animal survival by fat emulsion in liver transplant rats. Colloids Surf B Biointerfaces. 2007; 54:25–32. 17157486 10.1016/j.colsurfb.2006.05.017

[57] Morimoto Y, Kamiike W, Nishida T, Hatanaka N, Shimizu S, Huang TP, et al Improvement of rat liver graft function by insulin administration to donor. Gastroenterology. 1996; 111:1071–1080. 8831603 10.1016/s0016-5085(96)70076-8

[58] Song S, Shen X, Tang Y, Wang Z, Guo W, Ding G, et al Sinomenine pretreatment attenuates cold ischemia/reperfusion injury in rats: the role of heme oxygenase-1. Int Immunopharmacol. 2010; 10:679–684. 10.1016/j.intimp.2010.03.011 20353835

[59] Liang R, Bruns H, Kincius M, Lin T, Ludwig J, Dei-Anane G, et al Danshen protects liver grafts from ischemia/reperfusion injury in experimental liver transplantation in rats. Transpl Int. 2009; 22:1100–1109. 10.1111/j.1432-2277.2009.00925.x 19663939

[60] Chen T, Cheng M, Yuan Z, Zhou S, Yu Z. Protective role of Shenfu on ischemia-reperfusion injury of rat liver grafts. Transplant Proc. 2012; 44:978–981. 10.1016/j.transproceed.2012.03.052 22564601

[61] Zhu WH, Leng XS, Zhu JY. Effect of Shenfu injection on ischemia-reperfusion injury of rat liver graft. Hepatobiliary Pancreat Dis Int. 2006; 5:205–209. 16698576

[62] Zhu X, Qiu Y, Shi M, Ding Y. Matrine protects sinusoidal endothelial cells from cold ischemia and reperfusion injury in rat orthotopic liver transplantation. Ann Clin Lab Sci. 2003; 33:216–225. 12817627

[63] Zhu XH, Qiu YD, Shi MK, Wu B, Zheng XG, Ding YT. Effect of matrine on cold ischemia and reperfusion injury of sinusoidal endothelial cells in rat orthotopic liver transplantation. Acta Pharmacol Sin. 2003; 24:169–174. 12546726

[64] Tarrab E, Huet PM, Brault A, Rocheleau B, Laurens M, Crenesse D. Cyclosporin-A does not prevent cold ischemia/reperfusion injury of rat livers. J Surg Res. 2012; 175:333–342. 10.1016/j.jss.2011.04.018 21696775

[65] Chen LP, Zhang QH, Chen G, Qian YY, Shi BY, Dong JH. Rapamycin inhibits cholangiocyte regeneration by blocking interleukin-6-induced activation of signal transducer and activator of transcription 3 after liver transplantation. Liver Transpl. 2010; 16:204–214. 10.1002/lt.21985 20104495

[66] Gao W, Washington MK, Bentley RC, Clavien PA. Antiangiogenic agents protect liver sinusoidal lining cells from cold preservation injury in rat liver transplantation. Gastroenterology. 1997; 113:1692–1700. 9352874 10.1053/gast.1997.v113.pm9352874

[67] Xu HS, Rosenlof LK, Pruett TL, Jones RS. Prostaglandin E1 increases survival with extended anhepatic phase during liver transplantation. Ann Surg. 1994; 220:53–58. 8024359 10.1097/00000658-199407000-00009 PMC1234287

[68] Schemmer P, Schoonhoven R, Swenberg JA, Bunzendahl H, Thurman RG. Gentle in situ liver manipulation during organ harvest decreases survival after rat liver transplantation: role of Kupffer cells. Transplantation. 1998; 65:1015–1020. 9583858 10.1097/00007890-199804270-00001

[69] Higgins JP, Thompson SG, Deeks JJ, Altman DG. Measuring inconsistency in meta-analyses. Bmj. 2003; 327:557–560. 12958120 10.1136/bmj.327.7414.557 PMC192859

[70] Kotsch K, Ulrich F, Reutzel-Selke A, Pascher A, Faber W, Warnick P, et al Methylprednisolone therapy in deceased donors reduces inflammation in the donor liver and improves outcome after liver transplantation: a prospective randomized controlled trial. Ann Surg. 2008; 248:1042–1050. 10.1097/SLA.0b013e318190e70c 19092349

[71] Amatschek S, Wilflingseder J, Pones M, Kainz A, Bodingbauer M, Muhlbacher F, et al The effect of steroid pretreatment of deceased organ donors on liver allograft function: a blinded randomized placebo-controlled trial. J Hepatol. 2012; 56:1305–1309. 10.1016/j.jhep.2012.01.020 22326464 PMC3355301

[72] Baskin-Bey ES, Washburn K, Feng S, Oltersdorf T, Shapiro D, Huyghe M, et al Clinical Trial of the Pan-Caspase Inhibitor, IDN-6556, in Human Liver Preservation Injury. Am J Transplant. 2007; 7:218–225. 17227570 10.1111/j.1600-6143.2006.01595.x

[73] Busuttil RW, Lipshutz GS, Kupiec-Weglinski JW, Ponthieux S, Gjertson DW, Cheadle C, et al rPSGL-Ig for improvement of early liver allograft function: a double-blind, placebo-controlled, single-center phase II study. Am J Transplant. 2011; 11:786–797. 10.1111/j.1600-6143.2011.03441.x 21401865

[74] Weigand MA, Plachky J, Thies JC, Spies-Martin D, Otto G, Martin E, et al N-acetylcysteine attenuates the increase in alpha-glutathione S-transferase and circulating ICAM-1 and VCAM-1 after reperfusion in humans undergoing liver transplantation. Transplantation. 2001; 72:694–698. 11544433 10.1097/00007890-200108270-00023

[75] Hilmi IA, Peng Z, Planinsic RM, Damian D, Dai F, Tyurina YY, et al N-acetylcysteine does not prevent hepatorenal ischaemia-reperfusion injury in patients undergoing orthotopic liver transplantation. Nephrol Dial Transplant. 2010; 25:2328–2333. 10.1093/ndt/gfq077 20179007

[76] Yamanaka N, Takaya Y, Oriyama T, Furukawa K, Tanaka T, Tanaka W, et al Hepatoprotective effect of a nonselective endothelin receptor antagonist (TAK-044) in the transplanted liver. J Surg Res. 1997; 70:156–160. 9245565 10.1006/jsre.1997.5116

[77] Valero R, Garcia-Valdecasas JC, Net M, Beltran J, Ordi J, Gonzalez FX, et al L-arginine reduces liver and biliary tract damage after liver transplantation from non-heart-beating donor pigs. Transplantation. 2000; 70:730–737. 11003349 10.1097/00007890-200009150-00004

[78] Manika A, Trinh T, Lagace G, Dugas MA, Proulx F, Lepage G, et al N-acetylcysteine in pig liver transplantation form non-heart-beating donors. Transplantation. 1999; 68:327–330. 10459534 10.1097/00007890-199908150-00002

[79] Uhlmann D, Gaebel G, Armann B, Ludwig S, Hess J, Pietsch UC, et al Attenuation of proinflammatory gene expression and microcirculatory disturbances by endothelin_A_ receptor blockade after orthotopic liver transplantation in pigs. Surgery. 2006; 139:61–72. 16364719 10.1016/j.surg.2005.07.006

[80] Yokoyama I, Negita M, Kobayashi T, Hayashi S, Hachisuka T, Sato E, et al Beneficial effect of donor pretreatment with thromboxane A_2_ synthase inhibitor on the graft survival in pig liver transplantation. J Surg Res. 1996; 60:232–238. 8592420 10.1006/jsre.1996.0036

[81] Takada Y, Boudjema K, Jaeck D, Bel-Haouari M, Doghmi M, Chenard MP, et al Effects of platelet-activating factor antagonist on preservation/reperfusion injury of the graft in porcine orthotopic liver transplantation. Transplantation. 1995; 59:10–16. 7839408 10.1097/00007890-199501150-00003

[82] Aguilar-Melero P, Luque A, Machuca MM, Obanos M, Navarrete R, Rodriquez-Garcia IC, et al Cardiotrophin-1 reduces ischemia/reperfusion injury during liver transplant. J Surg Res. 2013; 181:E83–91. 10.1016/j.jss.2012.07.046 22906559

[83] Menger MD, Vollmar B. Pathomechanisms of ischemia-reperfusion injury as the basis for novel preventive strategies: is it time for the introduction of pleiotropic compounds? Transplant Proc. 2007; 39:485–488. 17362764 10.1016/j.transproceed.2007.01.022

[84] Monbaliu D, Vekemans K, Hoekstra H, Vaahtera L, Libbrecht L, Derveaux K, et al Multifactorial biological modulation of warm ischemia reperfusion injury in liver transplantation from non-heart-beating donors eliminates primary nonfunction and reduces bile salt toxicity. Ann Surg. 2009; 250:808–817. 10.1097/SLA.0b013e3181bdd787 19826248

[85] Abu-Amara M, Yang SY, Tapuria N, Fuller B, Davidson B, Seifalian A. Liver ischemia/reperfusion injury: processes in inflammatory networks—a review. Liver Transpl. 2010; 16:1016–1032. 10.1002/lt.22117 20818739

[86] McCormack L, Dutkowski P, El-Badry AM, Clavien PA. Liver transplantation using fatty livers: always feasible? J Hepatol. 2011; 54:1055–1062. 10.1016/j.jhep.2010.11.004 21145846

[87] Mendes-Braz M, Elias-Miro M, Jimenez-Castro MB, Casillas-Ramirez A, Ramalho FS, Peralta C. The current state of knowledge of hepatic ischemia-reperfusion injury based on its study in experimental models. J Biomed Biotechnol. 2012; 2012:298657 10.1155/2012/298657 22649277 PMC3357607

[88] Fondevila C, Shen XD, Duarte S, Busuttil RW, Coito AJ. Cytoprotective effects of a cyclic RGD peptide in steatotic liver cold ischemia and reperfusion injury. Am J Transplant. 2009; 9:2240–2250. 10.1111/j.1600-6143.2009.02759.x 19681824 PMC2981149

[89] Schmeding M, Rademacher S, Boas-Knoop S, Roecken C, Lendeckel U, Neuhaus P, et al rHuEPo reduces ischemia-reperfusion injury and improves survival after transplantation of fatty livers in rats. Transplantation. 2010; 89:161–168. 10.1097/TP.0b013e3181c425fd 20098278

[90] Moore C, Shen XD, Fondevila C, Coito AJ. Fibronectin-alpha4beta1 integrin interactions modulate p42/44 MAPK phosphorylation in steatotic liver cold ischemia-reperfusion injury. Transplant Proc. 2005; 37:432–434. 15808667 10.1016/j.transproceed.2004.12.206

[91] Amersi F, Shen XD, Moore C, Melinek J, Busuttil RW, Kupiec-Weglinski JW, et al Fibronectin-alpha 4 beta 1 integrin-mediated blockade protects genetically fat Zucker rat livers from ischemia/reperfusion injury. Am J Pathol. 2003; 162:1229–1239. 12651615 10.1016/s0002-9440(10)63919-3 PMC1851218

[92] Zhai Y, Busuttil RW, Kupiec-Weglinski JW. Liver ischemia and reperfusion injury: new insights into mechanisms of innate-adaptive immune-mediated tissue inflammation. Am J Transplant. 2011; 11:1563–1569. 10.1111/j.1600-6143.2011.03579.x 21668640 PMC3658307

